# The Influence of Form- and Meaning-Based Predictions on Cortical Speech Processing Under Challenging Listening Conditions: A MEG Study

**DOI:** 10.3389/fnins.2020.573254

**Published:** 2020-09-25

**Authors:** Carine Signoret, Lau M. Andersen, Örjan Dahlström, Rina Blomberg, Daniel Lundqvist, Mary Rudner, Jerker Rönnberg

**Affiliations:** ^1^Linnaeus Centre HEAD, Swedish Institute for Disability Research, Department of Behavioural Sciences and Learning, Linköping University, Linköping, Sweden; ^2^The National Research Facility for Magnetoencephalography, Department of Clinical Neuroscience, Karolinska Institutet, Solna, Sweden; ^3^Center of Functionally Integrative Neuroscience, Institute of Clinical Medicine, Aarhus University, Aarhus, Denmark

**Keywords:** speech perception, MEG, predictability, working memory, semantic knowledge, phonological knowledge

## Abstract

Under adverse listening conditions, prior linguistic knowledge about the form (i.e., phonology) and meaning (i.e., semantics) help us to predict what an interlocutor is about to say. Previous research has shown that accurate predictions of incoming speech increase speech intelligibility, and that semantic predictions enhance the perceptual clarity of degraded speech even when exact phonological predictions are possible. In addition, working memory (WM) is thought to have specific influence over anticipatory mechanisms by actively maintaining and updating the relevance of predicted vs. unpredicted speech inputs. However, the relative impact on speech processing of deviations from expectations related to form and meaning is incompletely understood. Here, we use MEG to investigate the cortical temporal processing of deviations from the expected form and meaning of final words during sentence processing. Our overall aim was to observe how deviations from the expected form and meaning modulate cortical speech processing under adverse listening conditions and investigate the degree to which this is associated with WM capacity. Results indicated that different types of deviations are processed differently in the auditory N400 and Mismatch Negativity (MMN) components. In particular, MMN was sensitive to the type of deviation (form or meaning) whereas the N400 was sensitive to the magnitude of the deviation rather than its type. WM capacity was associated with the ability to process phonological incoming information and semantic integration.

## Highlights

- Mismatch Negativity amplitude reflects the difficulty in phonological sensory perception.
- Preference for phonological information is observed in the left auditory cortex during sentence processing.
- Unrelated speech elicits larger N400 amplitudes than partially related speech (at semantic or phonological level) under adverse listening conditions.
- N400 effects appear to be more associated with the strength of deviation rather than the type of deviations.
- Working Memory plays a critical role in rejecting deviant stimuli and integrating expected ones.

## Introduction

The predictive brain hypothesis (Friston, 2009; Clark, 2013) describes the brain as an anticipatory organ that can generate predictions about the causal structure of the external world, based on the top-down influence of knowledge stored in long-term memory (Bar, 2007; Winkler et al., 2009; Friston, 2012). At the neural level, this is made anatomically possible by plastic corticopetal-corticofugal loops through which changes in activity at higher levels of the brain affect neural coding at lower levels of the brain including subcortical nuclei (Khalfa et al., 2001). In the domain of language comprehension, this predictive mechanism is thought to be crucial given that the speed and perceived ease with which complex speech signals are processed are influenced by the extent to which linguistic and contextual predictions have been preactivated (for a review, see Federmeier, 2007). Even though it has been observed in several studies that predictions can be generated at multiple levels (e.g., phonological, semantic) during language comprehension (for a review, see Kuperberg and Jaeger, 2016), it remains unclear whether deviations from expectations at different levels have a different impact on speech processing (for a review, see Nieuwland, 2019).

Numerous studies have shown that predictions about the form (i.e., phonology) and the meaning (i.e., semantics) of speech increase both its intelligibility (e.g., Miller et al., 1951; Davis and Johnsrude, 2007; Zekveld et al., 2011, 2013) and its perceptual clarity (Wild et al., 2012; Signoret et al., 2018; Signoret and Rudner, 2019). This facilitative effect could explain the enhanced perception of a speech event for which we already have knowledge stored in long-term memory – a phenomenon that leads to improved speech detection at a phonological level (see the “speech detection effect” in Signoret et al., 2011), better speech recognition at a lexical level (see the “word detection effect” in Signoret et al., 2011), and facilitated speech categorization at a semantic level (Daltrozzo et al., 2011; Rönnberg et al., 2019). Additionally, predictions about form and meaning have been shown to have an additive and independent facilitative effect on speech perception in that the meaning can still enhance the perceptual clarity of degraded speech even when total reliance on the form is possible (Signoret et al., 2018; Signoret and Rudner, 2019), suggesting that predictions about the form and the meaning could have different kinds of impact on neural speech processing.

### Meaning-Based Prediction Effects on Speech Processing

Several studies have indicated that meaning-based predictions play an important role in speech perception (for a review, see Van Petten and Luka, 2012), especially under adverse listening conditions (Obleser et al., 2007; Sheldon et al., 2008). It is even proposed that predictions about meaning have a stronger impact than predictions about form (see for example Ito et al., 2016). Indeed, recent behavioral results showed a facilitative effect of meaning-based predictions on speech comprehension and learning, but no effect of form-based predictions (see Experiments 1 and 2 in Corps and Rabagliati, 2020). Meaning-based predictions were also shown to be more robust than form-based predictions in a visual word experiment monitoring eye fixations (Ito et al., 2018). Participants fixated more often on picture targets and meaning-related pictures than on form-related or unrelated pictures after hearing sentences in which the final word was correctly expected. These behavioral observations were corroborated at a neural level with effects indexed by the N400 component, which is an evoked potential originally observed in EEG studies typically between 200 and 600 ms after stimulus onset (for a review, see Kutas and Hillyard, 1980; Kutas and Federmeier, 2011) in a distributed network including at least the left posterior part of the middle temporal gyrus (Brouwer and Hoeks, 2013). This component is modulated by the processing of meaning-based predictions where larger N400 amplitudes are observed in response to unexpected or less expected in a sentence than in response to highly expected words (see, for instance, Lau et al., 2009, 2013; Obleser and Kotz, 2010; Wang et al., 2012; Maess et al., 2016). In an EEG study investigating the temporal decay of meaning- and form-based predictions of final words in a sentence reading task (Ito et al., 2016), N400 amplitudes were larger for unrelated (i.e., deviant) stimuli than for stimuli whose meaning could be predicted, irrespective of the time allowed to generate the prediction. Moreover, N400 amplitudes were also larger for unrelated stimuli than for stimuli whose form could be predicted, but only when participants had a long time (i.e., 700 ms) to predict the final word, suggesting that meaning-based predictions could be generated faster than form-based predictions.

### Form-Based Prediction Effects on Speech Processing

Considering that knowledge-based predictions pre-activate representations about the form of an upcoming word (DeLong et al., 2005), it is likely that form-based predictions can bias processing to a limited set of phonological combinations (Ylinen et al., 2016). Although Nieuwland et al. (2018) were unable to replicate the N400 effect demonstrated in the study by DeLong et al. (2005), Nieuwland (2019) suggested that pre-activation of form is apparent in earlier brain responses. This hypothesis is in line with previous results showing that the perceived clarity of speech was greater when contingent on form-based predictions rather than meaning-based predictions, especially under adverse listening conditions (see Signoret et al., 2018; Signoret and Rudner, 2019). The difference in speech processing between form- and meaning-based predictions might then be observed on early neural activity, such as in the Mismatch Negativity (MMN) amplitudes. MMN effects are elicited by any deviation to standard, expected events, and reflects an automatic expression of change detection in neural predictions with regard to incoming auditory stimuli (for a review, see Näätänen et al., 2007). MMN amplitude modulation has been observed for example in phoneme discrimination (Näätänen et al., 1997) and localized to the auditory cortex (Poeppel et al., 1997). The MMN is reported to have larger amplitude for unexpected than expected events at a mean latency of about 160–170 ms (Schwade et al., 2017) and is most prominent in the left hemisphere (Shestakova et al., 2002). The MMN effect is thus considered as a viable index of predictive coding (Friston, 2012) and useful for the study of form-based representations in the brain.

### The Role of Working-Memory in Speech Processing

Current models of language understanding such as the Ease-of-Language Understanding (ELU) model (Rönnberg et al., 2008, 2013, 2019; in press), emphasize the integration of stimulus-driven and knowledge-based processes (McClelland and Elman, 1986; Hickok and Poeppel, 2007) when processing speech under adverse listening conditions. Such conditions are regularly encountered in everyday life situations where the perceived quality of speech signals can be affected by external factors in the form of background noise (e.g., in supermarkets, train stations, or classrooms) and signal distortion (e.g., phone calls) or by internal factors such as hearing impairment. There is an inverse relationship between the quality of speech signal and reliance upon knowledge-based predictions (see, for instance, Rogers et al., 2012; Rönnberg et al., 2013; Peelle, 2018) such that, the more the speech signal is degraded, the more the brain needs to rely on knowledge stored in long-term memory to predict the contents of incoming speech signals (Rönnberg et al., 2008). This reliance is reflected at a cognitive level through the engagement of working memory (WM), which is where knowledge-based predictions are likely to be formed and maintained during language understanding (Rönnberg et al., 2019).

Predicted speech events are processed with ease and make few demands on explicit WM processing (cf. prediction role, Rönnberg et al., 2019) while unpredicted or mispredicted (i.e., deviant) events require more explicit processing and load on WM capacity (cf. postdiction role, Rönnberg et al., 2019). A central role of WM is therefore to compare relevant knowledge-based contents active in memory with stimulus-driven processing for monitoring prediction error (Friston, 2012). This process may explain a variety of findings that correlate WM capacity with speech processing proficiency in adverse listening conditions where higher WM capacity is associated with better performance (Akeroyd, 2008; Besser et al., 2013; Rudner and Signoret, 2016). As phonology is proposed to be the bottleneck of lexical access in implicit and rapid information processing (Rönnberg et al., 2008, 2013, 2019), the reliance upon WM is probably greater when the form of the perceived content is deviant. At a neural level, WM capacity is plausibly reflected on the N400 component, with higher WM capacity associated with smaller N400 effects (Kim et al., 2018) in processing deviant compared to expected stimuli.

### Overview of the Current Study

The purpose of the present study was to explore how deviations from expectations modulate cortical speech processing under adverse listening conditions and how this affects speech processing. Based on the literature outlined above, we designed an experiment in which we explored how MMN and N400 components were affected by deviations from form- and meaning-based expectations. For this purpose, MEG recordings of ongoing brain activity were obtained while participants listened to familiar spoken sentences presented in background noise. We compared cortical responses to form- and/or meaning-based deviations from an expected final word in familiar sentences. In addition, we explored the extent to which processing of form and/or meaning deviations could be associated with WM capacity.

We experimentally varied the degree to which the final word of each sentence was related to the remainder of the sentence. The final word was either an *expected* word (i.e., the final word matched with prediction both in form and meaning, e.g., “The nearest doctor is so far, we’ll have to drive there in your car”) or a *deviant* word (see Table 1). Such deviants belonged to one of three categories*: meaning deviants* (deviating in meaning but related in form, e.g., “The nearest doctor is so far, we’ll have to drive there in your jar”), *form deviants* (deviating in form but related in meaning, e.g., “The nearest doctor is so far, we’ll have to drive there in your bus”), or *unrelated deviants* (deviating in both form and meaning, e.g., “The nearest doctor is so far, we’ll have to drive there in your plus”).

**TABLE 1:** Example of final word counterbalancing across the different experimental manipulations.

Sentence	Final word			
	*Hål*([ho:l]; hole)	*Vrål* ([vro:l]; roar)	*Grop* ([gru:p]; pit)	*Rop* ([ru:p]; cry)
*Grävskopan är byggd av stål, gräver fel och skapar __* (the excavator is built of steel, digs wrong and creates __)	Expected word	Meaning deviant	Form deviant	Unrelated deviant
*Härmar Tarzan efter mål, slag på bröstet blir till __* (the goal is to imitate Tarzan, punches to the chest and __)	Meaning deviant	Expected word	Unrelated deviant	Form deviant
*Snöret fastsatt med en knop, med en spade skapas __* (the cord fastened with a knot, with a shovel created a __)	Form deviant	Unrelated deviant	Expected word	Meaning deviant
*Högljudd skrikig antilop, skrämmer jägaren med __* (loud screaming antelope, scares the hunter with a __)	Unrelated deviant	Form deviant	Meaning deviant	Expected word

*The same final word was presented once in all experimental manipulations and therefore perfectly counterbalanced across all conditions. Sentences/words in the table are presented in italics in Swedish with a rough English translation in brackets.*

This experimental design utilizes the characteristics of the N400 and MMN response, where the literature has shown that magnitude of deviations from a prediction has a positive relationship to the magnitude of modulation of response components, so that an increase in response amplitude follows in an increase in the magnitude of deviation from predictions. Accordingly, we phrase our hypotheses from the perspective that the modulation magnitude of a particular response (such as MMN or N400) following a particular type of deviation (e.g., in form, meaning) will reveal whether that response component is sensitive or not to that particular type of deviation. Based on this general perspective, we hypothesize the following from our experimental design:

(1) Difference in amplitudes between expected and deviant final words: N400 as well as MMN amplitudes are larger for deviant than for expected final words under adverse listening conditions.
(2) Differences in amplitudes between the types of deviant: Unrelated deviants generate larger N400 effect compared to meaning deviants. Form deviants generate larger MMN effect compared to meaning deviants.
(3) Higher WM capacity is associated with better performance in processing final words, especially form deviants, and associated with smaller N400 effects.

## Materials and Methods

### Participants

Twenty-one young adults recruited from Linköping University participated in this study (thirteen males, mean age = 25.2, *SD* = 5.50). All participants were native Swedish speakers with no history of hearing impairment or neurological disease. For assessing hearing according to the American National Standards Institute (ANSI, 2004), the hearing thresholds at hearing frequencies 0.125–8 kHz were tested with an AC40 audiometer. Handedness was tested with the Edinburgh Handedness Inventory (Oldfield, 1971) and the safety for MEG inclusion was checked with a detailed questionnaire. After reading an information letter, all participants provided written informed consent to the study, which was conducted in accordance with the guidelines of the Declaration of Helsinki and approved by the Regional Ethics Committee in Linköping (2015/158-31). Participants were compensated with 500 SEK for their contribution to the study.

### Materials

#### Working Memory Test

To assess WM capacity participants completed a Swedish version of the Reading Span (RS) test (Daneman and Carpenter, 1980; Rönnberg et al., 1989). The RS test is composed of three-word sentences visually presented in blocks of 2–6 sentences. The sentences were presented word-by-word on a computer screen at a rate of one word per 800 ms. The sentences were grammatically correct, but half of the sentences made sense (such as “the tractor works well”) while the other half did not (such as “the fox reads poetry”). After reading each sentence, the participant had 5,000 ms to decide whether the sentence was absurd or not by pressing “yes” for absurd sentences and “no” for normal sentences. After a block, the participants were asked to recall either the first or the final words (determined randomly) of each sentence in their correct serial presentation order. There were two blocks per sentence list and the maximal available RS-score was 40 correctly recalled words.

#### Sentences

The sentence task consisted of two main conditions: *expected* vs. *deviant* final words. All final words in a sentence consisted of one syllable of three phonemes. In the expected condition, final words (48 words in total) were congruent with the remainder of the sentence both in form and meaning (see Supplementary Appendix), for example: *“The nearest doctor is so far, we’ll have to drive there in your car.”* Such final words were validated by 21 participants from Linköping University (12 males; mean age = 23.3 years, *SD* = 2.15 years), who had to end the sentence with the best adapted final word in a sentence completion test. These expected final words were evaluated by 10 other participants from Linköping University (5 males; mean age = 24.1 years, *SD* = 1.73 years) in an experiment in which they had to evaluate if the final word was the word they expected (yes/no response). The final words with the highest cloze probability scores (*M* = 0.95, *SD* = 0.003) were chosen as expected final words.

Final words in the deviant conditions belonged to one of three categories (of which there were 48 words in each): deviating in either form, meaning, or both. For the *meaning deviants*, violating predictions in meaning but related in form, the first phoneme was different from that of the expected final word, while the second and third phonemes were identical to the correct final word. Meaning deviants were also semantically absurd in relation to the first part of the sentence, for example: “*The nearest doctor is so far, we’ll have to drive there in your jar.”* For the *form deviants*, violating expectations in form but related in meaning, all the phonemes were different from the expected final word but the word was otherwise semantically similar to the predicted final word, for example: *“The nearest doctor is so far, we’ll have to drive there in your bus.”* For the *unrelated deviants*, all phonemes were different from the expected final word and also semantically absurd in relation to the first part of the sentence, for example: “*The nearest doctor is so far, we’ll have to drive there in your plus.”*

In order to get the same amount of *expected* vs. *deviant* final words within the experiment, the expected trials were repeated three times. A correct final word was thus presented in half of the trials (i.e., 144 trials), and a deviant final word was presented in the remaining half (i.e., 144 trials). In total, the same first part of the sentence was randomly repeated six times for each participant: three times with an expected final word and three times with a deviant final word (from, meaning, or unrelated deviants). As such, all 48 final words were used both as expected, form deviant, meaning deviant and unrelated deviant in accordance with the first part of the sentence within participant. In doing so, we were able to achieve perfect counterbalancing between the different experimental conditions (see Table 1). This design ensured that the observed effects could only be due to final word’s relationship with the first part of the sentence and not dependent upon word characteristics.

To load on WM during speech processing, sentence materials were presented in a background of continuous white noise. The loudness level of the speech material was set at 80% intelligibility (i.e., +1 dB SNR) for the first part of the sentences to give enough information to the listener for predicting the expected final word and 50% of intelligibility (i.e., −5 dB SNR) for the final words (see “words in context,” Figure 2 in Malmberg, 1970, p. 121) to load on WM and avoid ceiling effects. After the experiment, participants were asked to evaluate the sentence cloze for each presented final word with respect to its associated sentence on a five-point Liker scale (from 1 = not natural at all to 5 = very natural).

### Procedure

**FIGURE 1:**
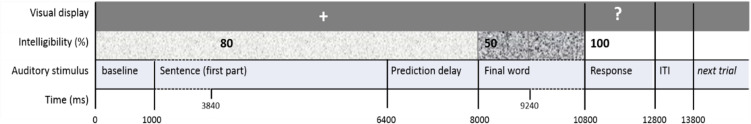
MEG experimental paradigm: at the beginning of each trial, a white cross fixation appeared on a black screen with a background of white noise. Sentences were presented at 80% intelligibility 1,000–3,840 ms after trial onset such that the first part of the sentence always ended 6,400 ms from the trial onset. After a prediction delay of 1,600 ms, the critical final word was presented at 50% intelligibility (by manipulating the loudness level of the final word and keeping the background noise level constant). Motor responses were collected 10,800 ms after trial onset, with the longest final word ending 9,240 ms from the trial onset.

Before the MEG experiment, the participants received instructions to read the 48 sentences pertaining to the expected condition (i.e., sentences ending with the predicted final words) at home, so they became familiar with the sentence material. After providing written informed consent to the study, participants were prepared for the MEG experiment. During the preparation, the experimenter checked that participants had read the sentence list at home and asked them to read the sentence list once again. This familiarization procedure was used to ensure that the participants knew the expected final word of each sentence, which was correct both in form and meaning. Throughout the experiment, participants listened to each sentence and assessed whether the final word was the “expected one,” i.e., the word appearing in the sentence list they had read (see experimental paradigm in Figure 1). Each trial began with a background of auditory white noise together with a white fixation cross visually centered on a black screen. As the first part of the sentences did not have identical durations, the onset of the sentence varied such that the offset of the first part of each sentence (i.e., before the presentation of the final word) occurred 6,400 ms after the beginning of the trial. This was followed by a delay period with a fixed duration of 1,600 ms, which was enough time to generate and maintain the knowledge-based linguistic predictions in WM. The final word of each sentence had an onset at 8,000 ms after the beginning of the trial. To ensure that motor activity would not be present in the MEG recording of linguistic processing, the participants had to delay the motor response to 2,800 ms after the onset of the final word. The participants were also instructed not to blink during the prediction delay or the presentation of the final word. The longest final word duration was 1,240 ms. When the background noise faded to silence, the fixation cross was replaced by the appraisal question *“Was the final word the correct one? (i.e., the one that you had read before).”* Participants had 2,000 ms to provide a motor response (by pressing yes/no buttons with the index or middle finger of the same hand, respectively). The response hand was counterbalanced across participants (i.e., 50% used the left hand and 50% used the right). Participants were instructed that they could blink at the time they responded. The inter-trial interval was 1,000 ms.

After the MEG experiment, a cognitive test battery including the RS test was administered to the participants who also filled in the sentence cloze evaluation. The testing after the MEG experiment took approximatively 40 min and the duration of the entire experiment, including breaks, was approximately 2 h.

### MEG Acquisition

The data were collected at The National Facility for Magnetoencephalography (NatMEG), Department of Clinical Neuroscience, Karolinska Institutet. Neuromagnetic data were recorded on the Elekta Neuromag TRIUX with a 306-channel whole-scalp system (sampling rate: 2,000 Hz; 0.1–660 Hz online bandpass filter) in a magnetically shielded, sound-proofed room (MSR; model AK3b from Vakuumschmelze GmbH, Hanau, Germany). Head position was monitored using four head-position indicators (HPI) coils together with subject-specific scalp measurements using a 3D digitizer (FASTRAK; Polhemus, Inc.) relative to three anatomical fiducial points: nasion, left pre-auricular, and right pre-auricular points. Ocular activity was monitored via bipolar horizontal and vertical electrooculography (EOG). Cardiac activity was monitored with bipolar electrocardiography (ECG), with electrodes attached below the left and right clavicle.

Stimulus presentation was synchronized with MEG recordings and behavioral responses using Presentation^®^ software (Version 18.1, Neurobehavioral Systems, Inc., Berkeley, CA). Auditory stimuli were presented through ear-tubes (model ADU1c, KAR Oy, Helsinki, Finland) to both ears. Visual instructions were projected onto a screen inside the magnetically shielded room (black background, white text). All 288 trials were presented with randomized order in one session including seven short breaks to allow participants to rest as long as they need and ask questions. During these breaks, participants were asked to evaluate their alertness on a scale (the Karolinska Sleepiness Scale, KSS; Akerstedt and Gillberg, 1990) between 1 (= extremely alert) to 9 (= very sleepy). Total recording time was approximately 1 h.

### MEG Preprocessing

Using MaxFilter v2.2 (Taulu and Simola, 2006), data from the MEG sensors (204 planar gradiometers and 102 magnetometers) were processed using temporal Signal Space Separation (tSSS) with a correlation limit of 0.95 and segment length of 10 s (Taulu et al., 2005; Taulu and Simola, 2006) to suppress noise sources, to compensate for head motion, and to reconstruct any bad sensors.

Subsequent processing was done in FieldTrip (Oostenveld et al., 2011) software implemented in MATLAB R2017b (The MathWorks, Inc., Natick, MA). The data segments were extracted from -200 ms before the final word presentation up to 1,500 ms after the onset of final word presentation. Only trials obtaining a correct answer (hits in correct condition and correct rejections in deviant conditions) were included. Segments containing system-related artifacts or muscular activity were identified based on signal variance. Identified segments were inspected visually and rejected if contamination with artifacts was confirmed. The remaining data were subsequently resampled at 300 Hz, lowpass-filtered below 40 Hz and baseline corrected by demeaning using the mean activity in the 200 ms leading up to the stimulation. Subsequently, independent component analysis (ICA) was performed (Makeig et al., 1996). Components explaining horizontal and vertical eye movements, eye blinks, and ECG were discarded based on visual inspection. On average, 1.85 components were excluded per participant. Sensor-level time series were reconstructed from the remaining components. After preprocessing, visual inspection of all the remaining segments was performed and the number of remaining trials varied from 134 to 236 per participant (on average 174.65 ± 35.16). The minimum number of remaining trials per conditions was 23 (out of 48) and was evaluated as enough to be included in the analysis. Timelocked analyses were then used to calculate the average responses for each participant, so-called event-related fields (ERF) for correct and deviant conditions and then for each deviant condition (form, meaning, and unrelated) separately.

### Statistical Analyses

#### Behavioral Performance

Behavioral analysis was conducted with Statistica analysis software (v.13; Hill and Lewicki, 2005). Signal Detection Theory (Green and Swets, 1974; Macmillan and Creelman, 2005) was used to analyses final word assessments. Hits were defined as participants answering “yes” when the expected final word was presented, and false alarms were defined as participants answering “yes” when deviant final word was presented. Correct rejections were defined as participants answering “no” when a deviant final word was presented, and omissions were defined as participants answering “no” when an expected final word was presented. We expected to obtain 50% hits (i.e., answering “yes” in the expected condition) as the intelligibility level was set to 50%. More interesting was to investigate whether deviants were identified as deviants. The *d*-prime measure was used to assess task performance, of which a high *d*-prime value corresponded to high task performance. A single *d*-prime score was obtained for each deviant type (form-related, meaning-related, and unrelated) and the variance in *d*-prime scores were compared by way of a within-subject ANOVA. Reaction times related to each deviant type were also investigated with a within-subject ANOVA. Differences in cloze scores of the final words in the post-experiment evaluation were also analyzed by way of a within-subject ANOVA on the factor Final Word, including all cloze conditions (correct, meaning-related, form-related, and unrelated). To highlight the involvement of WM capacity, Spearman correlations were calculated for WM capacity (i.e., RS scores) and false alarm percentage as well as the mean amplitude of N400 components for each deviant type (minus expected condition) on cluster showing significant differences. An alpha level of 0.05 was used as a significance level.

#### MEG Sensor-Level Analysis

The sensor-level analysis was performed on gradiometers and magnetometers on all epoch lengths (i.e., 0–1,500 ms) with a non-parametric cluster-based permutation statistical test (Maris and Oostenveld, 2007) to highlight **(1) processing differences between expected and deviant final words**. A two-sided paired *t*-test (“cfg.statistics = ft_statfun_depsamplesT”) was used for the generation of clusters with a threshold of 5% (“cfg.alpha = 0.05”). The likelihood of these clusters under the null hypothesis that the data is exchangeable were investigated using Monte-Carlo-randomizations (“cfg.method = ‘montecarlo”’, “cfg.numrandomization = 1,000,” “cfg.correctm = cluster”). The same procedure was used to highlight **(2) processing differences between the type of deviants**. The sensor-level analysis was also run on gradiometers and magnetometers between the different deviant conditions (i.e., form, meaning and unrelated deviants) focusing on later components such as the auditory N400 component (i.e., 200–600 ms post stimulus), but also on early responses such as the MMN (i.e., 120–200, focusing on the peak at about 160 ms and not overlapping with later effects). Grand-averaged ERFs were calculated for sensors that were part of the clusters found in the cluster-based permutation analysis.

#### Head-Modeling and Dipole Analysis

To localize which areas are involved in the differences observed between form and meaning deviants, source analysis was planned. Since gradiometers have a better signal-to-noise ratio on the Elekta TRIUX system, source modeling was based on data from the MEG gradiometer sensors. Head-modeling was performed using a whole-brain 3D volume from the Centre for Medical Image Science and Visualization (CMIV) at Linköping University, Sweden. The T1-weighted anatomical image was acquired using a Philips Ingenia 3.0 Tesla MRI scanner with a standard eight-element head coil. The following pulse sequence parameters were used: voxel sized of 1 × 1 × 1 mm^3^, TR = 25 ms, TE = 4.6 ms, 175 sagittal slices.

The first step in source modeling is to create a forward model indicating how sources in the brain connect to the sensors in the sensor array. To do this, the MR image and MEG sensor array were co-registered using a two-step procedure. First, the three fiducial points were found on the MR image (rough alignment). Afterward, an iterative closest points (ICP) algorithm was used to optimize the co-registration by minimizing the distance between the digitized head points (nasion, left pre-auricular, and right pre-auricular points) and the head surface. The co-registered image was subsequently segmented intro brain, skull, and scalp tissue. From the brain compartment a surface mesh was created, from which a single compartment volume conductor was created. The volume conductor indicates how magnetic fields spread for sources inside it. A source space was created by creating a regular grid of sources centered on the volume conductor. For each of the sources inside the volume conductor, a lead field was estimated, indicating how each source would be seen by each of the sensors.

The second step was to do the inverse modeling, estimating which source configuration best explained the sensor activity pattern on the sensors. For the early component (i.e., MMN, 120–200 ms), we chose to do a symmetrical dipole fit, fitting two dipoles at the same time under the assumption that they were symmetrical around the *x*-axis, i.e., ear-to-ear. This thus assumes two focal sources in the brain – which fits well with the expectation that there should be bilateral activity in the auditory cortices at such early latencies (Shahin et al., 2007). However, for the later component (i.e., N400, 200–600 ms), such dipole analysis was not performed since this component is observed in a distributed network (see, for example, Maess et al., 2006). The two dipoles were fitted for the activity in the time window of interest. A grid search was used, going through sources one by one, to find the optimal starting position. Subsequently, gradient descent was used to optimize the dipole on six parameters, i.e., the *xyz*-parameters of the position of the dipole and the *xyz*-parameters of its moment. The optimization finished when the difference between the sensor activity pattern produced by the two fitted dipoles and the actual sensor activity pattern could not be reduced any further. We fitted the two dipoles based on the gradiometer data and all the four conditions collapsed. To get the time courses for each condition separately, separate dipoles were estimated with the position fixed, just estimating the *xyz*-parameters of the moment using gradient descent.

## Results

### Behavioral Results

#### Sentence Experiment

**FIGURE 2:**
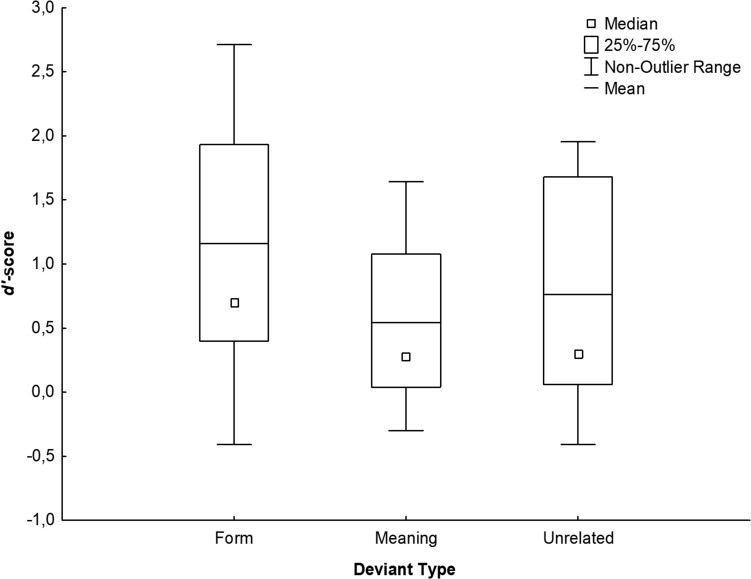
*d*′-scores for deviant final words (meaning, form and unrelated deviants). Higher *d*′-scores indicated better performance.

Overall performance on the recognition task was 64.32% (*SD* = 13.40). As expected, the performance (i.e., hits) was 50.33% (*SD* = 20.95) for the expected final words. In the deviant conditions, the performance (i.e., correct rejections) was higher: 69.74% (*SD* = 7.02) for meaning deviants, 85.12% (*SD* = 10.02) for form deviants and 75.75% (*SD* = 11.62) for unrelated deviants. Reaction times were significantly longer for meaning (*M* = 502.01 ms, *SD* = 136.79 ms) than for form (*M* = 461.96 ms, *SE* = 115.21 ms) deviants while no significant difference was observed between these two conditions and the unrelated deviants (*M* = 482.34 ms, *SE* = 131.56 ms; all *p*s > 0.45). Using *d’-*scores, the ANOVA revealed a main effect of Deviant Type [*F*(2, 40) = 44.11; *p* < 0.001; partial *η^2^* = 0.688] showing higher *d*′-scores for form (*d*′ = 1.16; *SE* = 0.21) than unrelated (*d*′ = 0.76; *SE* = 0.19) deviants, which had also higher *d*′-scores compared to meaning deviants (*d*′ = 0.54; *SE* = 0.14) (all *p*s < 0.006, Bonferroni corrected, see Figure 2).

#### Post-experiment Evaluation of Sentences

The post-experiment evaluation of sentence cloze revealed a significant main effect of the Final word [*F*(3, 60) = 155.67; *p* < 0.001] which showed higher cloze scores for the expected final word (*M* = 4.81; *SE* = 0.04) than form (*M* = 2.52; *SE* = 0.23), meaning (*M* = 1.77; *SE* = 0.13) or unrelated (*M* = 1.34; *SE* = 0.07) deviants. *Post hoc* analysis (Bonferroni corrected) showed significantly higher cloze scores for expected rather than form deviant word (*p* < 0.001), and for form rather than meaning (*p* < 0.001) or unrelated (*p* < 0.001) deviants. No statistically significant difference was observed between meaning and unrelated deviants (*p* = 0.098). These findings confirm that final words pertaining to the expected condition had the most natural sentence cloze and that meaning deviant or unrelated final words were judged as less natural than form deviant final words.

### Cortical Responses

Data from one participant was not included in the analysis because of too much movement (∼6 cm from origin), reducing the group to 20 participants (12 males, mean age = 25.4 years, *SD* = 5.6 years).

#### Differences Between Expected and Deviant Final Word Processing

**FIGURE 3:**
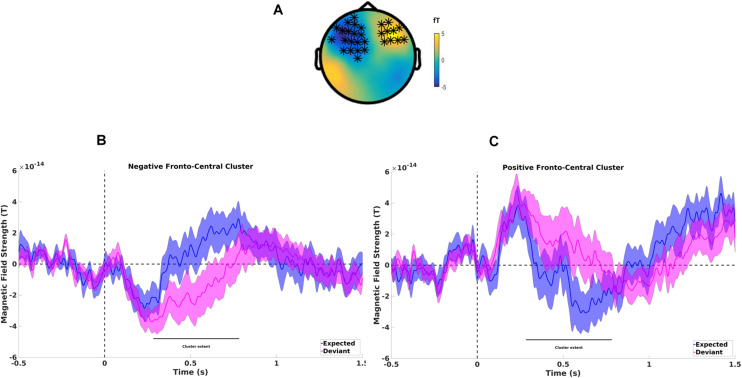
**(A)** Difference topography between expected and deviant last words showing a negative cluster found on left fronto-central sensors and a positive cluster on right frontal sensors. **(B)** Mean magnetometer activity for deviant (magenta line) and expected (blue line) final words for left fronto-central sensors of the negative cluster. Shaded areas represent pooled standard errors of the mean. **(C)** Mean magnetometer activity for deviant (magenta line) and expected (blue line) final words for left fronto-central sensors of the positive cluster. Shaded areas represent pooled standard errors of the mean.

Over the entire epoch length, the cluster-based permutation test indicated that there was a significant difference between ERFs related to word processing of expected and deviant words (see Figure 3A). A negative cluster most pronounced over left frontocentral magnetometers extended from approximately 283–783 ms (*p* < 0.002, see Figure 3B) while a positive cluster most pronounced over right frontal sensors extended from approximately 260–813 ms (*p* < 0.004, see Figure 3C), reflecting N400 effects on frontal sensors. Analysis on gradiometers showed comparable results with a positive cluster extended from 240 to 680 ms (*p* < 0.002) while a negative cluster extended from 260 to 840 ms (*p* < 0.004), localized predominantly over left sensors, and also over right fronto-central sensors. These findings extend results previously observed with higher N400 amplitudes for deviant vs. expected final words (Maess et al., 2016) from clear speech to adverse listening conditions.

#### Differences Between Deviant Final Word Processing

##### N400 effects

**FIGURE 4:**
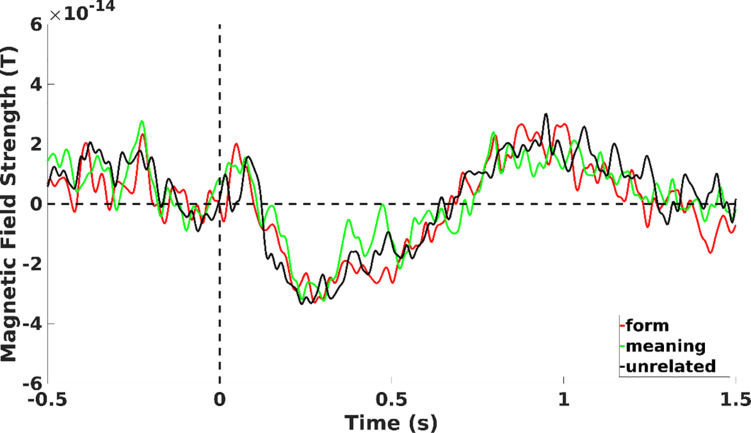
Mean magnetometer activity for meaning (green line), form (red line), and unrelated (black line) deviants on left fronto-central sensors of the negative cluster of Figure 3B.

Testing for N400 effects between the type of deviants (see Figure 4 showing mean magnetometer activity for each deviant type in the negative cluster reported for the entire epoch length), the cluster-based permutation test revealed a significant difference between unrelated deviants and the other types of deviants. A positive cluster over left temporal sensors revealed a larger N400 amplitude to unrelated vs. form deviants both for magnetometers (*p* = 0.048) and gradiometers (*p* = 0.002). Similarly, a larger N400 amplitude was found for unrelated compared to meaning deviants, although this difference was statistically significant only for magnetometers (*p* = 024). However, no significant difference was observed between form and meaning deviants (*p* > 0.05).

##### MMN effects

**FIGURE 5:**
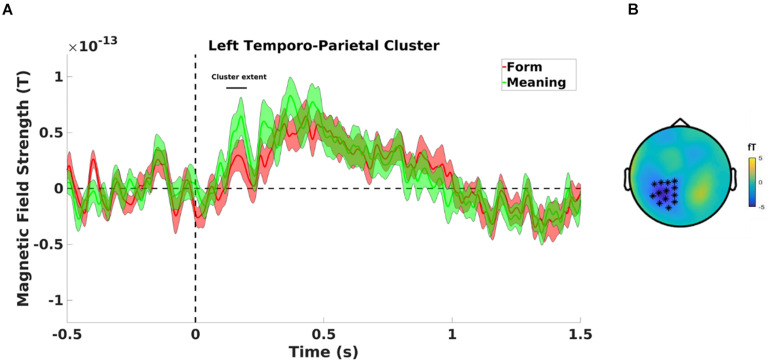
**(A)** Mean magnetometer activity for meaning deviant (green line) and form deviant (red line) on left temporo-parietal sensors, significantly different between 120 and 200 ms. Shaded areas are standard errors of the mean. **(B)** A negative cluster is found on left parietal sensors.

Testing for an MMN effect between the type of deviants, the cluster-based permutation test revealed a significant difference between form deviants and meaning deviants (see Figure 5A) in a negative cluster over left parietal magnetometer (*p* = 0.020, see Figure 5B) and left fronto-temporo-parietal gradiometer (*p* = 0.006) sensors. The cluster-based permutation test also showed a significant difference between meaning and unrelated deviants in a positive cluster over left temporo-parietal magnetometer (*p* = 0.014) and right middle parietal gradiometer (*p* = 0.020) sensors, revealing higher activity for meaning than unrelated deviants. No significant difference was observed between form and unrelated deviants (*p* > 0.05).

**FIGURE 6:**
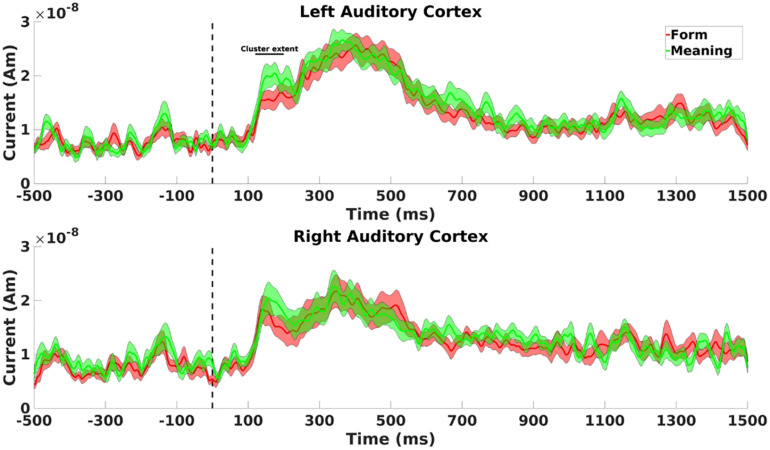
Cluster extent refers to Figure 6. **(Upper panel)** Dipole activity in the left auditory cortex with higher response to meaning (green line) than form (red line) deviants between 120 and 200 ms. **(Lower panel)** Dipole activity in the right auditory cortex for meaning deviants (green line) and form deviants (red line) showed no significant difference between 120 and 200 ms. Shaded areas represent pooled standard errors of the mean.

To localize the observed differences at sensor level in MMN amplitudes between form and meaning deviants, we used dipole analysis on gradiometers to model these responses at the anatomical source level. In 17 out of 20 participants, the results showed a bilateral dipole activity (120–200 ms) in the auditory cortex. On average for the whole-time window (120–200 ms), the dipole in the left hemisphere showed a significantly higher response amplitude following meaning compared to form deviants (*t*_(16)_ = −3.34; *p* = 0.004, see Figure 6, upper panel). No such difference was found for the dipole in the right hemisphere (*t*_(16)_ = −1.16; *p* = 0.264, see Figure 6, lower panel); and nor was a significant general difference between the dipoles in the left and right hemispheres (*t*_(16)_ = 1.29; *p* = 0.216).

### Working Memory Performance

**FIGURE 7:**
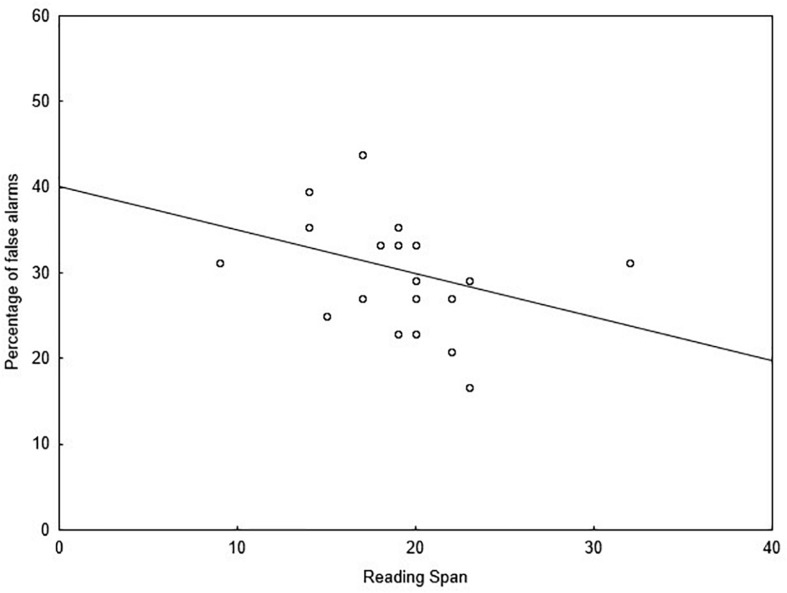
Scatterplot between the percentage of false alarms to the meaning deviants and RS-scores, showing significant Spearman’s rho correlation (*p* = 0.025; *y* = 40.07 –0.51*x).

One participant did not want to complete the WM test, reducing the group to 20 participants for behavioral data and 19 participants for MEG data. A significant correlation was observed between RS scores (*M* = 19, *SD* = 4.6) and false alarms for meaning deviants (*r*_s_ = −0.499; *p* = 0.025), but not for form deviants or unrelated deviants. This negative correlation indicated that participants with higher WM capacity experienced fewer false alarms (i.e., better performance) when processing meaning deviants which were rhyming with the expected final word (see Figure 7).

Testing for correlation between WM capacity and the mean amplitude of the N400 effects for each deviant type (minus expected condition) in significant clusters, RS scores were negatively associated with the N400 effect for meaning deviant [*r*_s_ = −0.622; *t*_(__19__)_ = −3.27; *p* = 0.004] but not to form deviant [*r*_s_ = 0.093; *t*_(__19__)_ = 0.38; *p* = 0.706] or unrelated [*r*_s_ = −0.002; *t*_(__19__)_ = −011; *p* = 0.991] final words in the positive cluster. This negative correlation indicated that participants with higher WM capacity had smaller N400 effects in response to meaning deviants compared to participants with lower WM capacity. No other significant correlation was found.

## Discussion

The present study investigated how cortical processing of degraded speech is affected when either/or both form- and meaning-based predictions about the incoming speech are violated. Participants familiarized themselves with the sentence material corresponding to the expected final word before testing. This allowed us to observe participant’s neural responses to prediction deviations by replacing final words of the familiar sentence with final words that were deviating to the first part of the sentence either in form, meaning, or both. Results showed that, under adverse listening conditions, meaning deviants elicited higher false alarm rate and larger neural activity in the left auditory cortex compared to form deviants, suggesting that meaning deviants were more difficult to process. Moreover, deviant final words evoked larger N400 amplitudes than expected final words, but no significant difference in N400 amplitude was found between final words that deviated in form and those that deviated in meaning. WM also appeared to play a significant role in the processing of final words, as higher WM scores were associated with better rejections and smaller N400 effects for meaning deviants.

### Behavioral Results and Limitations

Final words were presented in a background of white noise at a level of 50% intelligibility to induce adverse listening conditions loading on WM. This was confirmed by the performance level of the correct condition in which participants recognized the expected final word in relation to the pre-familiarized material in 50% of cases. Under deviant conditions, performance levels were much higher (77% overall across types of deviant), indicating that it was easier to reject the final word when it did not match knowledge-based predictions than to accept it when it did. However, correct rejections proved harder to make for meaning deviants than form deviants, so that participants responded slower and made more errors when processing meaning deviants compared to form or unrelated deviants. Having in mind that the meaning deviants are semantically incorrect but phonologically related to the expected final word, this result indicates that performance is lower when the final word rhymes with the expected final word. However, it is worth mentioning here that although the meaning deviants were phonologically related to the expected final word, they did not exactly match the expected final word on phonology. This could have induced difficulties in phonological processing. Interestingly, the rate of false alarms to meaning deviants was negatively associated with WM capacity. As a lower false alarm rate reflects better task performance, this result suggests that individuals with greater WM capacity were less likely to incorrectly classify final words phonologically related to the expected word as correct. In other words, individuals with greater WM capacity are less susceptible to phonological lures when listening to speech under challenging conditions (for a discussion see p.2 in Rudner et al., 2019). Furthermore, participants with higher WM capacity had smaller N400 effects in response to final words with deviant meaning compared to participants with lower WM capacity, indicating that the processing of phonologically related final words requires less neural resources for listeners with higher WM capacity. Plausibly, this finding indicates that WM is involved in the phonological analysis of the unfolding speech. However, this finding is limited by the experimental context of the study, which differs from everyday listening condition in the sense that listeners knew the sentences in advance (which is unlikely in everyday language comprehension). What is remarkable however is that WM was specifically involved in phonological processing but not semantic processing of the final words of a known sentence, which is in accordance with the assumption about lexical access being mediated by phonology in implicit and rapid information processing (see Rönnberg et al., 2008, 2013, 2019).

According to the ELU model (Rönnberg et al., 2008, 2013), knowledge-based predictions are held in WM until they have served their purpose. In the current experiment, knowledge-based predictions required both phonological and semantic knowledge to determine whether the final word in the sentence was the expected target. These findings suggest that WM capacity sets a limit for the retention of semantic information required to reject a phonologically matching word when listening to speech under adverse conditions. It might be suggested that WM capacity was particularly involved in the processing of phonological matching due to the specific design of our sentence materials. Given that for every sentence, the expected final word of the second clause always rhymed with the ending word of the first clause, and that a prediction delay of 1,600 ms was added between the first part of the sentence and the final word, we may have created a task-related bias toward phonology. WM involvement, in this case, may reflect the active maintenance of the rhyming sound which was possible to generate due to the prediction delay (see Ito et al., 2016). Initially, the rhyming design was intended to restrain the number of possible candidates for the final word to only one. Consequently, however, this phonological dimension may have resulted in greater difficulty to correctly identify and reject phonologically related final words. Nonetheless, when evaluating the sentence cloze with all the four possible final words (i.e., expected, form deviant, meaning deviant or unrelated deviant), it clearly appears that participants preferred (i.e., rated with higher scores) final words that matched the semantic context of the sentence over final words that phonologically rhymed with the sentence. Although form deviants made more sense than meaning deviants for participants when judging sentence cloze offline, meaning deviants still induced more recognition errors during online processing. This discrepancy in performance results between the online recognition task and the offline cloze task suggests that phonological predictions in noise may override semantic predictions under adverse listening conditions. Alternatively, these results may also be explained by the fact that the two tasks (i.e., the recognition task and the sentence cloze task) did not involve the same type of knowledge-based predictions. During the recognition task, participants may have relied more on their phonological knowledge in order to facilitate the processing of degraded speech as they have to listen to spoken sentences, whereas in the cloze task, participants needed only to rely upon their semantic knowledge to judge the naturalness of the sentence cloze.

### N400 Effects

Higher amplitude was observed over fronto-lateral sensors between 200 and 600 ms for deviant vs. expected conditions. This finding is in line with previous results showing that deviating words elicit larger N400 amplitudes than expected words (Maess et al., 2016) in quiet listening conditions. The present result extends this previous finding for speech perception *under adverse listening conditions* in which the intelligibility of the speech signal is compromised by background noise (see also Strauß et al., 2013 for other type of noise degradation). Participants were, therefore, more likely to rely on their knowledge-based predictions than on the word characteristics of the upcoming stimulus to perform the recognition task on the final word. Our findings suggest that pre-activations of linguistic representations associated with unfolding speech are necessary for efficient speech processing under adverse listening conditions.

Furthermore, form deviants elicited smaller N400 amplitudes than unrelated deviants (on both gradiometers and magnetometers), and meaning deviants also elicited smaller N400 amplitudes than unrelated deviants (only on magnetometers). These findings seem to contradict previous studies showing that meaning deviants and unrelated final words elicited similar N400 effects, especially in high-cloze sentences with prediction delays (Ito et al., 2016). But in our study, knowledge-based predictions had a strong phonological dimension due to the construction of the sentence material using a rhyming clause while the degree of semantic constraint was similar across conditions. The first part of the sentence was the same across all four experimental manipulations producing by consequence a similar constraint from knowledge-based predictions toward the upcoming final word, both on phonological and semantic characteristics. Then, participants had a long prediction delay (i.e., 1,600 ms) before hearing the final word, which was plenty of time for generating expectations at both phonological and semantic levels. This could explain why meaning and form deviants elicited smaller N400 effects than unrelated deviants that comprised both phonological and semantic anomalies. It should be noted that we have not observed differences between N400 effects related to form and meaning deviants which had only one type of deviation (either phonological or semantic). These results suggest that the N400 response likely reflects integration processes modulated by the strength of phonological or semantic deviation where accumulated deviation from semantic and phonological expectations results in larger N400 amplitudes. Thus, N400 effects are not reflecting prediction cost (as discussed in Luke and Christianson, 2016) but more probably the amount of matching between the predictions and the actual processed word (Kuperberg et al., 2020). This is also probably why WM capacity was associated with smaller N400 effects in processing final words with deviant meaning: it is possible that listeners with higher WM capacity processed meaning deviant more easily than participants with lower WM capacity. These results support the model proposed by Chen and Mirman (2012) which stipulates that phonological and semantic representations are activated simultaneously, and that precise phonological predictions will constrain the amount of all possible semantic predictions (Chen and Mirman, 2015). Taken together, these findings are in line with recent results suggesting that the N400 effects reflect a combination of prediction and integration processes (Nieuwland et al., 2020).

### Early Effects

The most interesting result of this study is the modulation of the MMN amplitudes by the type of prediction deviation since the observed MMN is related to early activity in the auditory cortex, and especially in the left hemisphere. In line with our hypothesis, higher amplitudes on left temporo-parietal sensors were observed for meaning compared to form deviants, both for gradiometers (between 120 and 200 ms), and for magnetometers (with peak activity around 180 ms). Additionally, these effects were localized to the left auditory cortex. This means that the left auditory cortex showed higher amplitudes in response to final words that are phonologically related to but semantically deviant from the expected final word. Because phonological language processing is usually left lateralized in the primary auditory cortex (Shestakova et al., 2002; Näätänen et al., 2007), this finding supports the notion that the left auditory cortex is preferentially prepared to respond to incoming phonological information. Since our study used the very same final words across sentences in all four experimental conditions, there were no differences in terms of acoustics or item characteristics between the different experimental conditions. Our carefully counterbalanced experimental design thus assured that any observed effect in this study was strictly due to the relationship between the final word and the knowledge-based expectations that were generated from the first part of the sentence. However, the downside of using such well-counterbalanced material is that the unrelated deviant also rhymed with the form deviant final words. This is probably the reason why we did not observe differences in early neural responses between unrelated deviants and form deviants. Instead, a significant difference in early cortical activity was observed between meaning deviants and unrelated deviants, which further supports the notion that the left auditory cortex has a preference for phonological information.

From the perspective of the predictive coding theory (Friston, 2009), MMN could be related to an early neural prediction error reflecting a discrepancy between the pre-activated neural memory trace of an expected stimulus and the phonological characteristics of the incoming speech sound. Thus, it could be proposed that phonological expectations primed the left auditory cortex via top-down influence. This result is in line with the assumptions proposed by the ELU model that considers phonology as the key for accessing the mental lexicon (Rönnberg et al., 2013, 2019) and there is accumulating evidence showing that phonological expectations can be observed in early cortical responses, before the N400 component (for a review, see Nieuwland et al., 2020). Nieuwland’s review (2020) shows that effects on the early time window referred as N200 (and that includes several components such as MMN or Phonological Mismatch Negativity) are increased by deviation from phonological predictions and are not differentiable from subsequent N400 effects. The author also concluded that further research is needed to disentangle N400 effects from earlier activity. In our study, we did not observe the same significant difference in MMN and N400 time windows: the difference in processing meaning and form deviants was significant for the MMN time window but not for the N400 time window, while the effect of processing unrelated deviants was significantly larger compared to the effect of processing meaning deviants only for the N400 time windows. The meaning deviants, which rhyme with the expected final words but have a different meaning, are also the deviants which induce most errors in the behavioral task, suggesting that they are the most difficult to separate from the expected final words. It is probably this difficulty in sensory processing that is reflected in early time windows, suggesting that the effects observed on the MMN time window are more likely related to sensory processing and focused on phonological processing in a comparison stage, while N400 effects are more likely related to cognitive processing in an integration stage, modulated by WM capacity in its postdiction role (Rönnberg et al., 2019).

## Conclusion

The present study aimed to investigate how the nature of knowledge-based predictions influence cortical speech processing under adverse listening conditions and whether this influence is associated with WM capacity. By manipulating the phonological and/or semantic relationship between a sentence and its final word, our results suggest that left auditory cortex may have been primed to preferentially respond to phonologically expected features of the incoming speech. In addition, WM appeared to play a role in the phonological processing of upcoming words. The results of this experiment provide support for an early neural mechanism responsible for comparing knowledge-based predictions with incoming speech signals. Taken together, these results suggest that the early effect could be related to the difficulty in sensory perception while the later effect could be related to integration processing in the sentence context.

## Data Availability Statement

The raw data supporting the conclusions of this article will be made available by the authors, without undue reservation, to any qualified researcher.

## Ethics Statement

The studies involving human participants were reviewed and approved by Regional Ethics Committee in Linköping (2015/158-31). The patients/participants provided their written informed consent to participate in this study.

## Author Contributions

CS, JR, MR, ÖD, and DL designed the experiment. CS and RB collected the data. CS and LA analyzed the data. All authors were involved in interpreting the results and writing the manuscript.

## Conflict of Interest

The authors declare that the research was conducted in the absence of any commercial or financial relationships that could be construed as a potential conflict of interest.
