# Supplementary material for: The Influence of Form- and Meaning-Based Predictions on Cortical Speech Processing Under Challenging Listening Conditions: A MEG Study
Source: Front Neurosci. 2020 Sep 25;14:573254. doi: 10.3389/fnins.2020.573254 (PMC7546411; doi:10.3389/fnins.2020.573254)
Supplement: Supplementary file 1 [file Data_Sheet_1.pdf]

SENTENCE LIST (IN SWEDISH)

List of the 48 correct sentences used in the Experiment. The correct final word of the sentence (in bold and underlined) is semantically correct and rhymes with the first clause of the sentence (underlined):

Massa ösregn staden fått, allt är nedsölat och **vått**

Trästatyer säljs av kock, men bara smala och ingen **tjock**

Alltid hundar omkring Max, även hemma har han **tax**

Andungarna har badardag, alla plaskar runt i **spad**

Boxar'n firade med rock, hade segrat med en **knock**

Varulven är farligt karg, både människa och **varg**

Häftig sittplats gjord i sol, skönt att sitta på en **stol**

Letade timmer fick en chock, hittade grenar men ingen **stock**

Strumpa, fot och hatt där opp', inte kantarell men kanske **sopp**

Vinner alltid eller hur, aldrig oflyt har han **tur**

Tuff och häftig gjord av tall, skönt att sitta på en **pall**

Välj en soft plats vid vår pool, ingen mesig men en **cool**

Inte alla men en del, gillar kramar, puss och **kel**

En rolig clown i lekparken skrek, det här var kul jag gillar **lek**

Jobbar med hundar i ur och skur, även hemma har han **djur**

Grävskopan är byggd av stål, gräver fel och skapar **hål**

Minikaminen sin värme spred, slukar tunna grenar men ingen **ved**

Minotauren var en lustig figur, både människa och **tjur**

Trots att han var alltför tjock, vann han enkelt vilken **chock**

Tid att vinna det är dags, om han vinner har han **flax**

Åker snowboard med tränad kropp, upp i luften ifrån **hopp**

Med en rullstol är det kamp, uppför trapp med hjälp av **ramp**

Timmerbilen i en krock, kvar blev grenar men ingen **stock**

Vattnet stannar i röret opp', för i vägen satt en **propp**

Tarzan han vill ej ha kramp, önskar gärna bo i **svamp**

Gula mannen sover sött, kudden gul men täcket **rött**

Badkaret läckte inte ett dugg, längst ned i botten satt en **plugg**

Törstig dricker efter lopp, och han använder en **kopp**

Säljer båtar och är glad, kunderna väntar fint i **rad**

Lammets släkting som ni vet, luktar illa och kallas **get**

Din träbock är för stor och sned, köp en smal men ingen **bred**

Högljudd skrikig antilop, skrämmer jägaren med **rop**

Alltid sist men ej ikväll, tänk han segra' vilken **skräll**

Trösta mig jag är så vek, kinden min den gillar **smek**

Snöret fastsatt med en knop, med en spade skapas **grop**

Härlig stranddag utan knott, stranden gul och vattnet **blått**

Äktenskapet blev så fel, otur i kärlek tur i **spel**

Simmade tills hon var trött, håret det blev mycket **blött**

Gubben inlåst i en bur, när man retar'n blir han **sur**

Vaktparaden simmar runt en ö, disciplinerat och i **kö**

Boxaren var alltför snäll, så han åkte på en **smäll**

Vintern den är kall och karg, retar mig så jag blir **arg**

Änder gillar inte snö, på sommaren de simmar fint i **sjö**

Härmar Tarzan efter mål, slag på bröstet blir till **vrål**

Välj en tuff plats i vår hall, ingen mesig men en **ball**

Alltid hungrig för liten rock, äter för mycket och kallas **tjock**

Hamstrar skräpmat ja jag vet, jag äter mycket och kallas **fet**

Har två horn och lever i flock, stångas gärna och kallas **bock**

Ville dricka efter tugg, gick och hämtade en **mugg**
